# Mental Health and Physical Activity of Female Higher Education Students during the COVID-19 Pandemic: A Comparative Cross-Sectional Study from Lithuania

**DOI:** 10.3390/ijerph19159725

**Published:** 2022-08-07

**Authors:** Marius Baranauskas, Ingrida Kupčiūnaitė, Rimantas Stukas

**Affiliations:** 1 Faculty of Biomedical Sciences, Panevėžys University of Applied Sciences, 35200 Panevėžys, Lithuania; 2 Institute of Health Sciences, Faculty of Medicine, Department of Public Health, Vilnius University, 01513 Vilnius, Lithuania

**Keywords:** mental health, mental disorders, psychological well-being, anxiety, depression, somatic complaints, physical activity, students, emerging adulthood

## Abstract

During emerging adulthood (EA), higher education medical students undergo a higher risk of anxiety and depression compared to the general population. The aim of this comparative cross-sectional study was to compare the proportions of three mental disorders, namely anxiety, depression and somatisation in terms of their symptoms and self-reported physical activity (PA) levels across the cohorts of biomedical and non-biomedical female students as well as to assess the association between the mental health outcomes and PA use. Between September 2021 and January 2022, a total of 1231 female higher education students aged between 18 and 29 years old were recruited for the study. Severe symptoms of anxiety and depression, as well as unexplained somatic complaints, were suffered by 51.9%, 11% and 23% of female students, respectively. Non-biomedical female students, compared to medicine and health sciences students, were more vulnerable due to the increased prevalence of negative mental health outcomes. The relationship between increased sports activity as a potential trigger for mental well-being and decreased severity of depressive symptoms was identified in the cohorts of both biomedical (adjusted odd ratio (OR_adj_) 0.4; 95% confidence interval (CI): 0.1–1.0) and non-biomedical (OR_adj_ 0.4; 95% CI: 0.2–0.9) female students. The current research highlights the importance of increasing sports activity by involving students in regular physical exercise of specific types for decreasing the severity of depressive symptoms in student-aged female populations.

## 1. Introduction

Globally, mental health disorders have increased by an average of 16% between 2005 and 2015 and will tend to rise further due to the increased exposure to risk factors in many countries [1]. Based on The Global Burden of Diseases, Injuries, and Risk Factors Study (GBD) in 2019, depression and anxiety are ranked amongst the 25 leading elements of the burden worldwide [2]. Depression is a widespread disease worldwide affecting 3.8% of the population, including 5% of adults [3]. Depression differs from usual mood alterations and brief emotional responses to challenges in day-to-day life [4]. However, in consonance with the American Psychiatric Association, anxiety disorders are also the most frequent type of mental health disorders and have an impact on nearly 30% of adults at some point in their lives [5]. Anxiety disorders comprise disorders that share features of enormous fear and anxiety and behaviour-related disturbances [6]. Additionally, both depression disorder and anxiety disorder as psychological reasons may have a close relationship with persistent, unexplained somatic symptoms [7]. Therefore, anxiety, depression issues and psychosomatic disorder related to physical symptoms without any form of physical irregularity are the most prevalent mental health problems worldwide, with dissimilarities in epidemiology existing across cultures and countries [8,9,10,11,12].

The potential triggers for increasing the development of mental disorders in the female gender and younger ages are related to changes in lifestyle, economic burden, place of residence and academic fears about post-graduation life [13,14]. More specifically, in agreement with GBD, the rates of depression and anxiety disorders were 50% higher in females compared to males [15,16], and they might pertain to potential risk factors, namely gender differences in social roles and responsibilities, cultural factors permitting greater expressiveness in women, lower socioeconomic status [17], lower threshold for seeking health care [18], lack of education and social support [19,20], traumatic experience, domestic violence, sexual abuse [21,22,23,24,25], beauty bullying [26,27] and cyber-bullying [28,29], gender discrimination [19], family dysfunction and negative family perceptions [30,31] and low estrogen levels [32]. Moreover, age periods related to mental health have been associated with both adolescence and emerging adulthood. In recent decades, the transition to a longer education time period and older age, with a commitment to stable relationships and having children, has led researchers to identify an important 18 to 29-year-old period of development called emerging adulthood (EA) [33]. The EA period was specified by the changes in love matters, work life and frequent housing adjustments [33,34]. The EA period was also linked to the main challenges related to building a stable life structure as well as completing education [33,34]. Some studies indicated that individuals have stable rates of depression over the earliest part of EA and the data regarding anxiety symptomatology are mixed [35,36]. However, screenings for mental health concerns must be carried out as early as possible during EA for several reasons. In particular, depressive and anxiety disorders emerging during adolescence increase the risk of developing both the same and other mental health problems during EA [14,37,38,39,40,41,42]. Secondly, various obstacles encountered in transitioning from education to profession can negatively alter the well-being of EA individuals; moreover, complicated transitions can result in mental health issues at a later age [43,44]. Consequently, monitoring mental health issue prevalence within the student-aged population, especially during EA, represents an important public health challenge. 

Students experience extensive demands in higher education, leading them to high levels of psychological distress that can result in serious disorders or the symptoms of depression, anxiety and somatisation [45]. It is well documented that students of medicine and health sciences attaining higher education undergo a higher risk of anxiety and depression compared to the general population [13,46,47,48,49,50,51]. Meanwhile, the overall proportion of depression disorder symptomatology amongst medicine and health science students varied from 1.4% to 73.5% [52,53], while the mean prevalence of anxiety symptoms was found to be 33.8% among medical students globally, which was substantially higher than the general population [48]. Nevertheless, additional attention must be focused on the fact that after the outbreak of the coronavirus disease (COVID-19) in Wuhan (Hubei Province, China) in December 2019, the severe acute respiratory syndrome coronavirus 2 (SARS-CoV-2) spread throughout the world. The World Health Organization (WHO) declared COVID-19 a global pandemic on 11 March 2020 and reported that since 9 July 2020 more than 12 million cases have been confirmed in more than 200 countries and regions [54]. To date, there have been more than 6,424,274 deaths associated with the disease globally [55]. During this time, from 3 January 2020 to 2 August 2022, in Lithuania there were 1,185,583 confirmed cases of COVID-19 with 9210 deaths reported to the WHO [56]. The COVID-19 pandemic has posed not only a higher risk of mortality from the SARS-CoV-2 infection but also public health issues associated with the symptoms of depression and anxiety worldwide [57,58,59,60]. According to the epidemiological data reported by Lithuanian scholars, it can be recognised that during the COVID-19 pandemic, the proportion of case-level depression and anxiety symptoms in the student-aged population increased to 11.1% and 46.6%, respectively [61]. It should also be emphasised that the COVID-19 pandemic has significantly influenced the lifestyle of higher education students when the Lithuanian government implemented effective quarantine measures and restricted the contact course in higher education institutions in order to contain the spread of the SARS-CoV-2 infection. Students experienced difficulties in maintaining both their personal relationships due to social distancing restrictions as well as the concentration of attention in the learning process [62]. Students have been affected by economic instability as a result of the disruption of on-campus employment. Suspended scientific research and internships were related to the decline in student competitiveness in the future labour market [63]. Thus, while the COVID-19 crisis continues, higher education students as EA individuals have entered the zone of higher risk for mental health issues [64,65,66]. 

A recently declared position paper on research superiorities for the field of mental health science concerning COVID-19 [67] stipulated the interdisciplinary development of innovative interventions to assure mental well-being by mechanistically based methods to enhance prosocial behaviour. In connection with this position statement, physical activity (PA) interventions were envisaged as a promising approach, taking into account that regular aerobic exercise may provide antidepressant and anxiolytic effects [68,69,70] and is capable of protecting the organism from the harmful effects of stress on physical and mental health [70]. However, decreased levels of PA were identified in the general population in many countries [71,72,73] during the COVID-19 pandemic crisis. Overall, despite the fact that PA has been recognised as an effective method to promote mental health, at present it still remains unclear how to conceptualise the association between PA use and specific symptoms of anxiety, and emerging depression across student-aged populations during EA. On the other hand, according to some pre-pandemic reports, there does not appear to be an additional benefit to mental health associated with meeting the WHO-recommended levels of activity [74]; also, it was revealed that there is inconsistent evidence regarding the association between the self-reported PA levels and depressive symptoms in students [75].

Basically, while many research studies have revealed that healthcare students may experience mental health problems such as depression, anxiety and somatic symptoms, only a few studies reported mixed results [50,51,76,77,78,79] or at the same time compared the pooled prevalence of these symptomologies between students of medicine and health sciences and students studying other disciplines of science. There are no unambiguous scientific reports published on the specific relationships between mental health well-being and PA loading during EA. Furthermore, although effective treatments for mental disorders have been reported, more than 75% of people in low- and middle-income countries are at a high risk of being unrecognised and receiving no treatment [80]. The situation in the Republic of Lithuania has been no exception, resulting in the emergence of barriers to effective mental health care depending on the social stigmatisation associated with mental disorders. Therefore, early diagnosis using questionnaires for screening the symptoms of mental disorders is highly recommended for healthcare providers to subsequently confirm the clinical diagnosis and offer necessary psychological treatments. Given that more women are affected by mental health disorders than men globally [4] and focusing on the existing gap in the availability of generalised results justifying a higher level of anxiety, depressive and somatic symptoms in biomedical students compared to those in non-biomedical students (exclusively in a cohort of females [76,77,78,79,81,82,83,84,85]), this article examined the following research questions (RQ): 

RQ1: To what extent do disparities exist in the proportions of case-level depressive and anxiety symptoms and clinically relevant somatic symptoms in the samples of female biomedical vs. non-biomedical students?

RQ2: To what degree do disparities endure in self-reported habitual PA levels across the cohorts of female biomedical and non-biomedical students?

RQ3: Does the severity of anxiety and depression symptoms have a relationship with the somatic complaints of female students?

RQ4: Does mental health well-being have an association with the use of PA?

In the light of the above, the paper was structured as follows. Section 2 provides a methodological framework describing the target population, study design, measures and statistical analysis methods. Section 3 displays the descriptive, frequency and regression analyses that led to obtaining evidence/information/data on how potential determinants such as anxiety symptoms and depressive symptoms may predict the somatic complaints in a cohort of female students. In Section 3, the association between the severity of the symptoms triggered by mental disorders and the PA use in the student-aged population was also assessed. In Section 4, an overview of the prevalence and incidence of symptoms of mental disorders, the disparities in mental health, the association between the somatic complaints and comorbid mental disorder symptoms and the relationship between PA use and the severity of depressive and anxiety symptoms are presented. The generalised current study findings and the implications for public health are detailed in Section 5.

## 2. Materials and Methods

### 2.1. Study Population, Area and Design

The study was conducted as a comparative cross-sectional study. The study included the data collected in the second half of 2021 during the fourth wave of the COVID-19 pandemic in the Republic of Lithuania, 16 months following the start of the COVID-19 pandemic announced by the WHO on 11 March 2020 [86]. The sample size was ascertained using the minimum number per group of students required for the study using the standard formula for the sample size in a comparative study and setting the study power at 90% [87]. The recruitment procedure of students of higher education institutions followed the method of simple random sampling. In September 2021, the participants were recruited through the websites of the 67 official Facebook groups administrated by 11 universities and 10 colleges in Lithuania. The administrators of the Facebook groups in different higher education institutions then provided the link through their own social network, Facebook, so that any student who accessed the site could complete the survey. 

**Figure 1:**
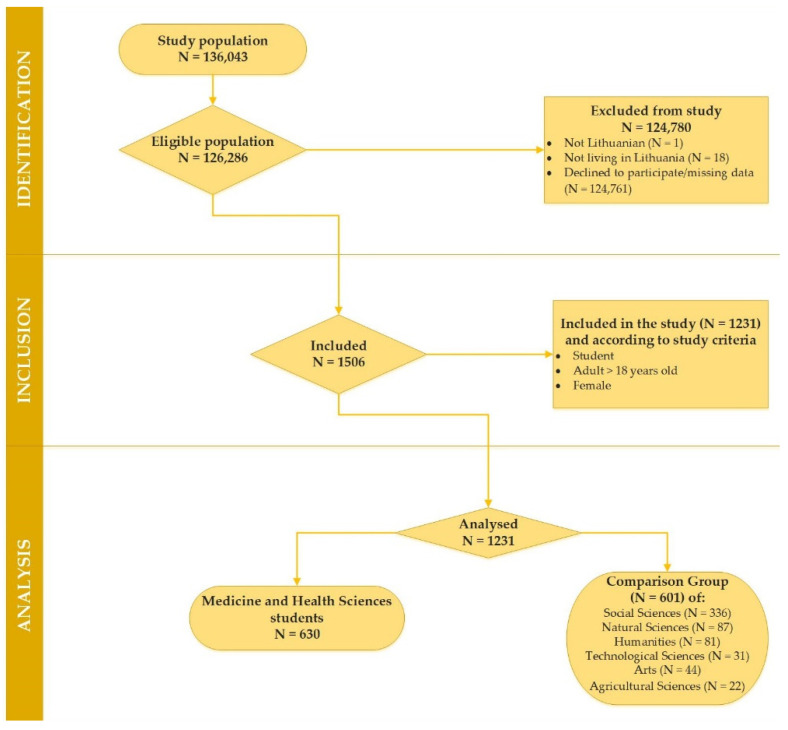
Consort flowchart depicting the reasons for the survey participants’ exclusion from the study.

A total of 126,286 higher education students were approached to participate in this non-experimental study. After the participants read a brief instruction describing the research and became interested in participating in the survey, they were asked to follow a link to the website with an online questionnaire. The questionnaire was included in an online survey (Apklausa, 204 version), and the web-based E-survey research application was used to collect information (https://apklausa.lt/private/forms/ [accessed on 9 September 2021]). Of the eligible population (N = 126,286), 124,780 students were excluded from the study due to a lack of entry criteria or declining to fill in the questionnaires. The data of female students (N = 1231) aged 18 to 29 (during EA) within January–June 2022 were included as well as analysed. A more detailed analysis of the study recruitment process is provided in Figure 1.

As identified in Table 1, the following stages of this comparative cross-sectional study data analysis related to the study population consisted of two groups: a target group comprised female students of medicine and health sciences (MHS) (N = 630) and a comparison group (CG) comprised female students of social sciences, natural sciences, technological sciences, agricultural sciences, humanities and arts (N = 601).

### 2.2. Measures

The study participants were surveyed on their sociodemographic characteristics. The data were collected on age; sex; branch of study (medicine and health sciences, natural sciences, social sciences, humanities, agricultural sciences, technological sciences or arts); year of academic study, further classified into 1st year, 2nd and 3rd year, 4–6th year; income (euros per month); place of residence, further categorized into those living alone in their own apartment, living alone in a rented apartment, living with parents, living with friends, living with relatives or living in the dormitory; marital status was further classified into married, single or divorced. 

This study also concentrated on the anxiety, depressive and somatic symptoms and habitual physical activity (PA), which were measured by three screening instruments elected from the investigations conducted by Baecke et al. [88], Kroenke et al. [89] and Zigmond and Snaith [90].

#### 2.2.1. The Hospital Anxiety and Depression Scale (HADS)

A validated Lithuanian version of HADS was used to assess the symptoms of anxiety and depression in a sample of students [91,92,93]. HADS consisted of a self-rating of 14 items, while two groups of 7 separate items described the two subscales, namely the subscale for anxiety (HADS-A) and the subscale for depression (HADS-D) [90]. The answer to each item on this scale was scored between 0 and 3 by taking into account the experience of the study participant over the past week. The possible cumulative score ranged from 0 to 21 points on both the HADS-A and HADS-D subscales. The HADS scale scores were categorized into groups that identified the risk of developing anxiety and depression: scores equal to or less than 7 recognized the absence of the symptoms of anxiety or depression, scores between 8 and 10 showed a borderline of these mental health disorders and scores equal to or above 11 assessed a high risk of developing a case of anxiety or depression [93].

#### 2.2.2. The Patient Health Questionnaire (PHQ-15)

A standardised version (in Lithuanian) of PHQ-15 (a 15-item scale) was used to measure the severity of the subjectively perceived somatic symptoms in this study’s participants over the past month [94,95,96]. The scale included a list of 15 common somatic symptoms. Each symptom was scored on a scale of 0 (“not bothered at all”), 1 (“bothered a little”) and 2 (“bothered a lot”) points. The sum of possible scores ranged from 0 to 30. The highest possible score for females was 30. The PHQ-15 does not maintain the diagnosis of whether the somatic symptoms are medically elucidated or unexplained. However, a high score on PHQ-15 scale was strongly related to physician-rated somatoform disorder symptom counts [97,98]. Thus, the total scores in the variations 0–4, 5–9, 10–14 and 15–30 on the PHQ-15 characterized the severity of somatic symptoms as “none”, “mild”, “moderate” and “severe (case)” [99].

#### 2.2.3. Baecke Physical Activity Questionnaire (BPAQ)

BPAQ [88] is demonstrated to have high reliability and accurate assessment of high intensity activity as well as low intensity activities [100,101,102]. Therefore, this instrument was used for evaluating PA and its three separate, distinct dimensions, namely work activity (“work index”), sports activity (“sport index”) and leisure-time activity (“leisure index”) in a sample of students over the last 12 months. This 16-item scale was previously translated into Lithuanian and has been used for epidemiological studies only [103,104]. The first 8-item subscale for work activity was related to occupation-related PA. As the target population was students, work activity was contemplated as the activity while studying. The second 4-item subscale for sport activity was associated with the practice of regular physical exercise of specific types, which was allocated into the three intensity levels according to the energy expenditure: light, moderate or vigorous activities. The duration and frequency (hours per week and months per year) for each activity were identified. The third 4-item subscale for leisure-time activity was correlated with non-sport-related activity, such as watching television (sedentary activity), walking, cycling and energy expenditure according to the number of minutes spent per day on these locomotive activities. The questions were asked on a five-point Likert scale. The measurements of PA were executed by calculating the sum of the scores acquired from three main domains of PAs. BPAQ allowed us interpretations of how the study participants were loading their PA.

### 2.3. Statistical Analysis

The statistical analysis was performed using SPSS V.25 for Windows (Armonk, NY, USA). The normality of variable distribution was tested by the Shapiro–Wilk *W* test. All categorical variables are presented as relative frequencies. Frequency analysis was used to designate the prevalence of symptomatology of mental disorders in the population under analysis on the testimony of its splitting into individual intervals in consonance with the severity of the selected mental problems.

The differences and correlations in variables (year of study, income, marital status, place of residence and categorized scores of HADS-A, HADS-D, PHQ-15) between groups (MHS students vs. CG) were assessed using Pearson’s χ^2^ test as well as using Cramer’s V (V) and phi (φ) correlation coefficients. A higher degree of the absolute value of the coefficient described a stronger relationship between the variables in contingency tables. The correlations above 0.4 were considered to be relatively strong and strong; the correlations between 0.2 and 0.4 were moderate; and those below 0.2 were weak. The critical level of the significance was considered as α = 0.05. 

Additionally, the central tendency measures (mean ± standard deviation (SD)) were used to reveal the gross scores of the data under analysis. Student’s *t* test (for equal variances) was used to assess the differences between the two categories. Cohen’s d (*d*) was also estimated for a better comparison of the effect size in identifying the branches of study. In conformity with Cohen [105], the results were observable as small effect size (0.2 ≤ *d* < 0.5), medium effect size (0.5 ≤ *d* < 0.8), and large effect size (0.8 ≤ *d* < 1.3). 

Multiple linear regression analyses were used to assess the association between the severity of somatic complaints (PHQ-15 score) and the intensity of both anxiety (HADS-A score) and depression symptoms (HADS-D score). Meanwhile, the main analysis was assigned to the application of multiple logistic regression with a binary dependent variable in subgroups allocated to different branches of science. The dependent variables, namely anxiety symptoms (HADS-A, 11 > score ≥ 11), depressive symptoms (HADS-D, 11 > score ≥ 11) and somatic symptoms (PHQ-15, 16 > score ≥ 16), were adjusted to the dichotomous form (0–the absence and mild severity of a mental health problem (reference category), 1–higher severity of the symptoms of mental disorders at case level). The scores of independent variables, namely Baecke index (7.6 > score ≥ 7.6), sport index (2.5 > score ≥ 2.5), work index (2.4 > score ≥ 2.4) and leisure index (2.8 > score ≥ 2.8) were adjusted to the dichotomous form depending on cut-off points that were identified using the median values of both PA and the three main domains of PAs. All regression models were adjusted for age, marital status, housing and income. Goodness-of-fit of logistic regression models was assessed using the Nagelkerke R^2^ statistic.

## 3. Results

### 3.1. Descriptive and Frequency Analyses

Between September 2021 and January 2022, a total of 1231 female students were recruited in this non-experimental cross-sectional study. A representative sample consisted of 630 MHS female students and 601 study participants in the CG, namely students of the social, natural, technological and agricultural sciences and the humanities and arts. Mean age ± SD for the MHS group was 21.8 ± 3.8 years, while this was 21.4 ± 3.5 years for the CG. Table 2 shows more detailed demographic characteristics, including qualities, namely year of study, income, marital status and information on housing across MHS and CG cohorts.

**Figure 2:**
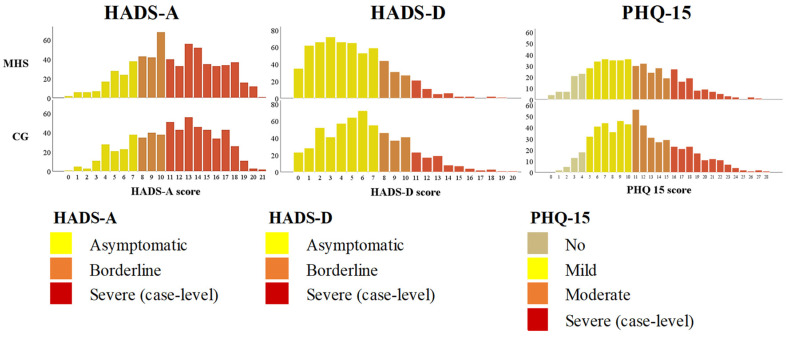
Distribution of HADS-A, HADS-D, PHQ-15 scores between the samples of female students of the groups of medicine and health sciences (MHS) and the comparison group (CG): natural sciences, social sciences, technological sciences, agricultural sciences, technological sciences, humanities and arts.

Figure 2 displays the distribution of the selected mental health concerns related to anxiety, depression and somatic symptoms among the MHS and CG female students. 

Overall, female students reported the most positive outcomes for anxiety (HADS-A) and somatisation (PHQ-15), and oppositely, the slightest positive outcomes were identified for depression symptoms (HADS-D). In terms of somatisation, a significantly higher PHQ-15 score was recognised in the CG (CG: mean = 11.5 ± 5.3 vs. MHS: mean = 10.6 ± 5.3; *d* = 0.2; *p* < 0.0001). CG female students also reported a significantly higher score for the two remaining mental problems, that is anxiety (HADS-A CG: mean = 11.7 ± 4.4 vs. MHS: mean = 9.6 ± 4.5; *d* = 0.4, *p* < 0.0001) and depression symptoms (HADS-D CG: mean = 6.4 ± 3.8 vs. MHS: mean = 5.2 ± 3.5; *d* = 0.3, *p* < 0.0001.

Table 2 presents the proportion of the most serious rates of mental disorders reported by MHS and CG respondents. Overall, the highest rates of mental health problems were found in 51.9% of female students with severe anxiety symptoms, 11% with severe depressive symptoms, and 21.3% with severe somatic complaints. Different distributions of the selected mental problems were recognized across female student groups. The proportion of case-level HADS severe anxiety and depression and the case-level PHQ-15 severe somatisation was significantly higher in the CG cohort when compared to the MHS group. In the CG, severe anxiety symptoms were identified in 59.6% of female students, case-level depression in 14.1% and severe somatisation in 22.5%. Meanwhile, in the MHS sample, 44.6% of female students reported severe anxiety symptoms, 7.9% of participants expressed severe depression, and 19.9% of female students experienced severe somatic symptoms.

Additionally, Table 2 shows the results of how study participants were loading their PA. While evaluating the PA of the female students in the areas of sports, leisure and work, it was found that average of the index score associated with the total Baecke PA score equaled 7.7 ± 1.3, the sport index score amounted to 2.5 ± 0.7, the leisure index score corresponded to 2.7 ± 0.6, and the work index score equaled 2.5 ± 0.6. 

Different distributions of PA loading were revealed in the samples of MHS and CG female students. The fact that female students representing MHS group in contrast to CG have been found to be more physically active was supported by the higher mean scores for total PA (7.9 ± 1.3 vs. 7.4 ± 1.3), sport (2.6 ± 0.7 vs. 2.3 ± 0.7), leisure (2.8 ± 0.6 vs. 2.7 ± 0.6) and work (2.6 ± 0.6 vs. 2.4 ± 0.6) indexes. Therefore, in terms of effect size (*d*), it can be affirmed that the higher rates for total, sport, leisure and work indexes of PA were established in the MHS student category, while the lower rate of PA loading scores were found in a sample of CG female students; thus, these rates could be assigned to small effect sizes (*d* = 0.4, *d* = 0.4, *d* = 0.2 and *d* = 0.3, respectively).

### 3.2. Regression Analyses

The results of the multivariate analysis are displayed in Table 3. Multivariate linear regressions were constructed to obtain how the determinants such as anxiety (HADS-A score) and depression (HADS-D score) may predict the somatic complaints (PHQ-15 score) in the samples of female biomedical and non-biomedical students. Multivariate linear regression models were adjusted for student age, marital status, housing information and income. 

After using a multivariate linear regression method, we revealed the following: if an increase of one score in the PHQ-15 scale is detected, there is also an increase in the severity of anxiety symptoms (HADS-A score) in the groups of both MHS and CG from 0.5 to 0.7 (β 0.6, *p* < 0.0001) and from 0.4 to 0.6 (β 0.5, *p* < 0.0001), respectively. Additionally, the association was established between the severity of somatic complaints and depressive symptoms in the samples of MHS (β 0.3; 95% confidence intervals (95% CI): 0.2–0.4; *p* < 0.0001) and CG (β 0.2; 95% CI: 0.04–0.3; *p* < 0.0001) female students.

Table 4 presents adjusted odds ratios (ORs_adj_) and 95% CIs for the multivariate logistic regression models. The best fitting multivariate logistic models adjusted for age and the demographic characteristics marital status, housing information and income were used to determine the associations of the selected mental health problems with PA loading in both samples of MHS and CG female students. 

Two logistic models evaluated the association between the depression scale scores and the PA index in the student-aged population. In case-level HADS severe depression, the ORs_adj_ for increased sports activity in the cohorts of MHS and CG female students were 0.4 (95% CI: 0.1–1) and 0.4 (95% CI: 0.2–0.9), respectively; thus, these results revealed the association between decreased depression symptomatology and increased sports activity related to the practice of regular physical exercise of specific types. However, the association between habitual PA and anxiety disorder symptomatology could not be explained by the total Baecke score or PA loading across the three separate, distinct dimensions, namely work activity, sports activity and leisure-time activity.

## 4. Discussion

### 4.1. The Incidence for Symptoms of Mental Disorders

Among the Lithuanian female students of medicine and health sciences, the proportions of clinically relevant anxiety and depressive symptoms were 44.6% and 7.9%, respectively. While comparing our study data with research reports derived from other countries, differences were observed. The prevalence of clinically relevant depressive symptoms in the cohorts of medical students during the COVID-19 pandemic was recognised as significantly higher in such countries as Russia [106], Jordan [107] and Greece [108] and ranged from 21.6% to 36.5%. A similar proportion of case-level HADS depression among medical students was found in the national cross-sectional studies involving France (9.1%) [109] and Palestine (14%) [110] compared to the rates we identified in Lithuania (7.9%). Meanwhile, the anxiety disorder symptomatology among the Lithuanian female biomedical and non-biomedical students (44.6% vs. 59.6%) we studied was significantly more intense compared to the pre-pandemic and mid-pandemic reports on the mental well-being state (in terms of anxiety) of medical students from other countries such as Greece [108], Russia [106], France [109], Pakistan [111], Malaysia [112], the United Kingdom (UK) [113], Portugal [78], Jordan [107], Palestine [110] and Bangladesh [114].

Our study found that low-level somatic complaints were very common among the student-aged population. However, a considerable proportion of high-level somatic symptoms related to severe somatisation disorder were identified in both biomedical and non-biomedical female students and equaled 22.5% and 19.9%, respectively. A similar, slightly lower proportion (14.5%) of severe somatic complaints experienced during the COVID-19 crisis was found among Lithuanian students by other scholars [115]. On the contrary, the proportion of severe somatic symptoms suffered by Czech (12.5%), Slovak (10.3%) [116] and Chinese (7.6%) [117] students during the COVID-19 pandemic was twice as low compared to our study data. 

### 4.2. Somatic Complaints and Comorbid Mental Disorders Symptoms

When persistent somatic symptoms may be evocative of mental health disorders, namely anxiety and depression [118,119], our study also found the association existing between somatic complaints and anxiety/depressive symptoms in both samples of Lithuanian female students. Similar results can be observed in other populations [120]. The relationship between somatic complaints and depressive symptoms has been well studied [121]. It is well documented that somatic symptoms in youths have been related to impaired physical and mental well-being and health [122]. Additionally, theories have been pursued to elucidate the mechanisms underlying somatic symptoms in EA individuals, such as the stress-leading process and its impact on pain and other complaints [123]. Thus, while somatic complaints in the student-age population may be related to depression and anxiety, nowadays the management of EA individuals, especially women with unexplained somatic symptoms, is still challenging [124]. Identification and psychological care may help to avoid somatisation disorder in terms of the full-blown form and long-term mental health problems. Hence, when student-aged females present with somatic complaints, mental health professionals must further consider screening for anxiety and depression symptomatology.

### 4.3. The Disparities in Mental Health

Another important topic of discussion is mental health inequalities in the samples of biomedical and non-biomedical students. Earlier studies have revealed the presence of specific triggers related to medical school which are consequently related to depressive symptoms. Potential factors associated with the high prevalence of psychosocial distress leading to greater expression of mental health disorder symptomatology in the segment of medical students have been identified in the scientific literature [125,126,127]. However, there is a shortage of research studies examining both depressive and anxiety symptoms among medical students and non-medical students. Only the results of studies carried out in Sweden [50], the United States, Canada [82], Germany [128], Portugal [78] and Jordan [79] have confirmed that medical students had significantly higher levels of depression and anxiety compared to non-medical students. Meanwhile, our study conducted in Lithuania, demonstrated controversial results and a significantly lower prevalence of the symptoms of mental disorders among medicine and health science students compared to non-biomedical students. Our study data coincided with the findings of comparative studies that were previously conducted in Lithuania [93], Israel [129], New Zealand [130] and Pakistan [111] and showed a lower intensity of subthreshold depressive and anxiety symptoms in medical students compared to non-medical students.

### 4.4. Physical Activity for Symptoms of Depression and Anxiety

Our study was conducted during the COVID-19 pandemic period when the requirements related to staying at home due to self-quarantine were correlated with an increased mental health burden and reduced habitual physical activity among residents [131]. PA as a provider of multipotent and powerful systemic neuroprotective effects was considered a potential protector of mental well-being during the quarantine regimen. More specifically, it was proposed that PA can reduce the severity of the symptoms of mental disorders by acting through various psychosocial and biological mechanisms, such as increasing the production of endorphins and a neurotrophic factor (BDNF) in the human body, improving the immune system or promoting self-esteem [120]. According to our study, the findings in a group of female medicine and health science students were associated with a more positive mental well-being and higher PA levels across all distinct dimensions such as work activity, sports activity and leisure-time activity compared to the same characteristics specified to female non-biomedical students. On the other hand, only the severity of depressive symptoms was associated with an increased sports activity pertaining to the involvement in regular physical exercise of specific types were displayed in the samples of both biomedical and non-biomedical students. These data were consistent with those revealed by a prior meta-analysis study, demonstrating that PA decreased the depressive symptoms with a medium effect [132]. By analogy, the findings of our study were also in line with those of a recent cross-sectional study stipulating that engaging in a moderate and high volume of exercise was associated with lower depression symptomatology in non-clinical adult individuals [133]. Nonetheless, even though our study did not support the findings related to the positive effects of PA on anxiety symptomatology reported by other scholars [132,133,134], it was consistent with some cross-sectional studies and randomised, controlled trials [135,136,137] and was able to indicate the absence of beneficial effects of PA on reducing anxiety symptoms.

However, the results derived from our study were unexpected since dose–response studies [138,139] proposed that more PA had a greater beneficial impact on mental health (in terms of both anxiety and depression). Although the biological development mechanisms for anxiety and depression have not been verified as identical, nevertheless, the high rate of comorbidity between these mental health problems was related to similar symptoms resulting in the incidence of both disorders. Thus, we hypothesised that there is still no clear evidence on how to conceptualise or define the mental health (in terms of anxiety and depression) benefits of PA as well as suggested that these hypotheses could serve as the research issues for future longitudinal studies in design in order to validate the causal relationships among these variables. 

### 4.5. Limitations of the Study

The first limitation of this study was related to the fact that the causal relationship between depression symptomatology and habitual PA should be elucidated with caution as this study was cross-sectional in design. Moreover, most preclinical studies evaluated whether PA can protect from future depressive symptoms and did not define the association between the type (i.e., mode) of PA and the mental health outcome [140,141]. Since the questionnaire we used to assess the PA of these subjects did not differentiate aerobic from anaerobic activities and the estimate of other variables referred to exercise direction, such as intensity, duration and frequency, these biases were considered as the third study limitation. Further, there is emerging evidence suggesting that the individuals engaging in both aerobic (i.e., moderate/vigorous physical activity) and muscle-strengthening activity protocols have the lowest odds ratio for depressive symptoms compared to those involved in only one program of regular physical exercise (either aerobic or anaerobic) [142]. Further experimental studies in research design must be conducted in order to assess the effect of PA characteristics such as intensity, duration and sport on mental well-being in student-aged populations. Future research must also focus on comparing the results related to mental health outcomes in EA individuals during the COVID-19 crisis with the research data received during the post-pandemic period. Additionally, other possible study limitations referred to the self-report nature of data collection in lieu of clinical diagnoses and some risk of skewing the results in fulfilment of the non-random sampling procedure. However, the selection of quota sampling was the most convenient option in the given circumstances of the COVID-19 crisis. The present study also corresponded to possible bias and error as it failed to assess the effect of pandemic preparedness measures on the onset of mental disorder symptoms and the impact of pandemic prevention management on exercise activities.

## 5. Conclusions

The present study showed that the proportion of case-level mental health problems such as anxiety and depression, including somatic complaints, was relatively high among female higher education students. The severe symptoms of anxiety and depression as well as unexplained somatic complaints were suffered by 51.9%, 11% and 23% of female students, respectively. Female non-biomedical students were more vulnerable due to the increased prevalence of negative mental health outcomes than those of medicine and health sciences. This study also confirmed the association between clinically relevant somatic complaints and the severity of other symptoms of comorbid mental disorders, and hence the screening for both depression and anxiety in female students during the student-aged period should additionally be considered.

Notwithstanding the fact that only female students of medicine and the health sciences were engaged in higher level physical activity, the findings of this study revealed the relationship between increased sports activity as a potential trigger for mental well-being and decreased severity of depressive symptoms in the cohorts of both biomedical and non-biomedical students. However, this study did not support the hypothesis that higher levels of physical activity play an important role in mediating between the variable related to the branch of study and the decreased severity of anxiety symptoms.

With regard to the generalised results of this study, the implications for public health should focus on necessary monitoring of mental health among emerging adulthood individuals and interventions for improving student-aged female populations’ mental health literacy as well as decreasing stigma [143,144]. The study also emphasises the importance of evolving effective student-centred programs aimed to develop positive coping skills and behaviours [145,146]. Additionally, the current research expands the scientific literature on the association between the use of physical activity and the severity of depressive symptoms during the pandemic period as well as highlights the importance of increasing sports activity concerning involvement in regular physical exercise of specific types for decreasing the severity of depressive symptoms in student-aged female populations. Eventually, it was well documented that sport performed in green spaces has an immune-strengthening efficiency effect provided by biogenic volatile compounds emitted by trees and might be beneficial for anxiety outcomes [147]. Consequently, potential determinants related to outdoor sports coupled with green sports may play a key role in the mental well-being of student-aged populations and serve as a direction for further research.

## Figures and Tables

**Table 1:** The composition of female student-aged population in the study.

Branch of Science	Eligible (N = 85,024)		Analysed (N = 1231)	
	N	%	N	%
Medicine and Health Sciences (MHS)	12,930	15.2	630	51.1
Comparison group (CG)	72,094	84.8	601	49.9
Social and Natural Sciences	53,048	73.5	423	70.3
Humanities and Arts	9304	12.9	125	20.8
Technological and Agricultural Sciences	9742	13.5	53	9

**Table 2:** Female students of medicine and health sciences by categorisation of demographic characteristics, habitual physical activity, somatic symptoms and symptomatology of anxiety and depression.

Variables	Medicine and Health Sciences (N = 630)		Comparison Group ^†^ (N = 601)		V ^a^/*d* ^b^	Total (N = 1231)	
	N	%	N	%		N	%
Age (yr), mean ± SD	21.8 ± 3.8		21.4 ± 3.5		0.1 ^b^	21.6 ± 3.7	
Year of study							
1st	226	48.4	241	51.6	0.2 ^b,^***	467	37.9
2nd	140	46.7	160	53.3		300	24.4
3rd	82	45.8	97	54.2		179	14.5
4–6th	182	63.9	103	36.1		285	23.2
Income (euros (€) per month)							
<200	213	46.6	243	53.4	0.04 ^b^	456	37
200–500	201	44.5	250	55.5		451	36.6
>500	146	45	178	55		324	26.3
Marital status							
Married	31	54.4	26	45.6	0.03 ^a^	57	4.6
Single	591	50.9	571	49.1		1162	94.4
Divorced	8	66.7	4	33.3		12	1
Housing							
In one‘s own apartment	76	58.9	53	41.1	0.1 ^a^	129	10.5
In a rented apartment	157	47.4	174	52.6		331	26.9
With parents	247	54.6	205	45.4		452	36.7
With friends	34	58.6	24	41.4		58	4.7
With relatives	10	58.8	7	41.2		17	1.4
In a dormitory	106	43.4	138	56.6		244	19.8
HADS-A score, mean ± SD	9.6 ± 4.5		11.7 ± 4.4			10.4 ± 4.5	
Asymptomatic (score ≤ 7)	220	62.9	130	37.1	0.4 ^b,^***	350	28.4
Borderline (score: 8–10)	129	53.3	113	46.7		242	19.7
Severe (case) (score ≥ 11)	281	44	358	56		639	51.9
HADS-D score, mean ± SD	5.2 ± 3.5		6.4 ± 3.8			5.8 ± 3.7	
Asymptomatic (score ≤ 7)	478	54.9	392	45.1	0.3 ^b,^***	870	70.7
Borderline (score: 8–10)	102	45.1	124	54.9		226	18.4
Severe (case) (score ≥ 11)	50	37	85	63		135	11
PHQ-15 score, mean ± SD	10.6 ± 5.3		11.5 ± 5.3			11.1 ± 5.3	
Asymptomatic (score < 7)	101	56.3	78	43.8	0.2 ^b,^***	179	14.6
Mild (score: 5–10)	198	45.6	235	54.4		433	35.2
Moderate (score: 11–14)	149	41.8	208	58.2		357	29
Severe (case) (score ≥ 15)	101	42.3	151	57.7		262	21.3
Baecke total score, mean ± SD	7.9 ± 1.3		7.4 ± 1.3		0.4 ^b,^***	7.7 ± 1.3	
Sport index score, mean ± SD	2.6 ± 0.7		2.3 ± 0.7		0.4 ^b,^***	2.5 ± 0.7	
Leisure index score, mean ± SD	2.8 ± 0.6		2.7 ± 0.6		0.2 ^b,^***	2.7 ± 0.6	
Work index score, mean ± SD	2.6 ± 0.6		2.4 ± 0.6		0.3 ^b,^***	2.5 ± 0.6	

^a^—Cramer’s V correlation coefficient (V); ^b^—the effect size (*d*); SD–standard deviation; ***—*p*-value < 0.001. Significant results are highlighted in bold. ^†^—comparison group (CG): natural sciences, social sciences, technological sciences, agricultural sciences, technological sciences, humanities and arts.

**Table 3:** Linear regression analyses with somatic symptoms (PHQ-15) as dependent variables.

PHQ-15 Score	Medicine and Health Sciences ^1^			Comparison Group ^2,†^		
	Β	95% CI	*p*	β	95% CI	*p*
HADS-A score	0.6	(0.5; 0.7)	<0.0001	0.5	(0.4; 0.6)	<0.0001
HADS-D score	0.3	(0.2; 0.4)	<0.0001	0.2	(0.04; 0.3)	0.007

The associations between somatic symptoms (PHQ-15 score) and HADS-A and HADS-D scores were estimated by controlling for student age and demographic characteristics, namely, marital status, housing information, income (adjusted for age, marital status, housing information and income). ^1^—F (6491) = 51.6, *p* < 0.0001, R^2^_adj_ = 0.38; ^2^—F (6592) = 35.9, *p* < 0.0001, R^2^_adj_ = 0.26; ^†^—comparison group (CG): natural sciences, social sciences, technological sciences, agricultural sciences, technological sciences, humanities and arts.

**Table 4:** Logistic regression analyses with symptoms of anxiety and depression (HADS-A and HADS-D) as dependent variables.

Variables	Baecke Index (Score)		Sport Index (Score)		Work Index (Score)		Leisure Index (Score)	
	β (SE)	OR_adj_ (95% CI)	β (SE)	OR_adj_ (95% CI)	β (SE)	OR_adj_ (95% CI)	β (SE)	OR_adj_ (95% CI)
Medicine and Health Sciences								
HADS-A score ≥ 11 ^1,a^	0.2 (0.3)	1.2 (0.6–2.4)	0.02 (0.3)	0.9 (0.6–1.7)	0.5 (0.3)	1.6 (1–2.6)	–0.4 (0.3)	0.7 (0.4–1.1)
HADS-D score ≥ 11 ^2,b^	–0.4 (0.6)	0.9 (0.2–3.1)	–1.1 (0.5) *	0.4 (0.1–1)	0.2 (0.4)	1.2 (0.5–2.8)	–0.1 (0.4)	0.9 (0.4–2)
Comparison group ^†^								
HADS-A score ≥ 11 ^3,c^	0.2 (0.3)	1.2 (0.6–2.2)	–0.1 (0.2)	0.9 (0.5–1.5)	0.2 (0.2)	1.2 (0.8–1.9)	0.1 (0.2)	1.1 (0.7–1.8)
HADS-D score ≥ 11 ^4,d^	–0.4 (0.4)	0.7 (0.3–1.6)	–0.9 (0.4) *	0.4 (0.2–0.9)	0.2 (0.4)	1.2 (0.6–2.4)	0.4 (0.3)	1.5 (0.8–2.7)

^†^—Natural sciences, social sciences, technological sciences, agricultural sciences, humanities and arts; OR_adj_—adjusted odds ratio (OR_adj_ = e^β^); 95% CI–95% confidence interval; SE–standard error; *—*p*-value < 0.05. Significant results are highlighted in bold. ^1^—reference category: HADS-A score ≤ 10 (asymptomatic and borderline anxiety); ^a^—Nagelkerke R^2^ = 0.32; the logistic regression model was adjusted for age, marital status, housing and income of medicine and health science students. ^2^—reference category: HADS-D score ≤ 10 (asymptomatic and borderline depression); ^b^—Nagelkerke R^2^ = 0.32, the logistic regression model was adjusted for age, housing and income of medicine and health science students; ^3^—reference category: HADS-A score ≤ 10 (asymptomatic and borderline anxiety); ^c^—Nagelkerke R^2^ = 0.32, the logistic regression model was adjusted for age, marital status and income of subjects in a comparison group ^†^. ^4^—reference category: HADS-D score ≤ 10 (asymptomatic and borderline depression); ^d^—Nagelkerke R^2^ = 0.32, the logistic regression model was adjusted for age and income of subjects in a comparison group ^†^.

## Data Availability

Data are available on request.
